# Overcoming Phosphorus Deficiency in West African Pearl Millet and *Sorghum* Production Systems: Promising Options for Crop Improvement

**DOI:** 10.3389/fpls.2016.01389

**Published:** 2016-09-23

**Authors:** Dorcus C. Gemenet, Willmar L. Leiser, Francesca Beggi, Ludger H. Herrmann, Vincent Vadez, Henry F. W. Rattunde, Eva Weltzien, Charles T. Hash, Andreas Buerkert, Bettina I. G. Haussmann

**Affiliations:** ^1^International Potato CentreLima, Peru; ^2^State Plant Breeding Institute, University of HohenheimStuttgart, Germany; ^3^Bioversity International-NASC ComplexNew Delhi, India; ^4^Institute of Soil Science and Land Evaluation, University of HohenheimStuttgart, Germany; ^5^International Crops Research Institute for the Semi-Arid TropicsPatancheru, India; ^6^University of Wisconsin-Madison, MadisonWI, USA; ^7^International Crops Research Institute for the Semi-Arid Tropics-MaliRemagen, Germany; ^8^International Crops Research Institute for the Semi-Arid TropicsNiamey, Niger; ^9^Organic Plant Production and Agroecosystems Research in the Tropics and Subtropics, University of KasselKassel, Germany; ^10^Institute of Plant Breeding, Seed Science and Population Genetics, University of HohenheimStuttgart, Germany

**Keywords:** phosphorus use efficiency, low-P tolerance, sorghum, pearl millet, sahel

## Abstract

West Africa (WA) is among the most food insecure regions. Rapid human population growth and stagnating crop yields greatly contribute to this fact. Poor soil fertility, especially low plant available phosphorus (P) is constraining food production in the region. P-fertilizer use in WA is among the lowest in the world due to inaccessibility and high prices, often unaffordable to resource-poor subsistence farmers. This article provides an overview of soil P-deficiency in WA and opportunities to overcome it by exploiting sorghum and pearl millet genetic diversity. The topic is examined from the perspectives of plant breeding, soil science, plant physiology, plant nutrition, and agronomy, thereby referring to recent results obtained in a joint interdisciplinary research project, and reported literature. Specific objectives are to summarize: (1) The global problem of P scarcity and how it will affect WA farmers; (2) Soil P dynamics in WA soils; (3) Plant responses to P deficiency; (4) Opportunities to breed for improved crop adaptation to P-limited conditions; (5) Challenges and trade-offs for improving sorghum and pearl millet adaptation to low-P conditions in WA; and (6) Systems approaches to address soil P-deficiency in WA. Sorghum and pearl millet in WA exhibit highly significant genetic variation for P-uptake efficiency, P-utilization efficiency, and grain yield under P-limited conditions indicating the possibility of breeding P-efficient varieties. Direct selection under P-limited conditions was more efficient than indirect selection under high-P conditions. Combining P-uptake and P-utilization efficiency is recommendable for WA to avoid further soil mining. Genomic regions responsible for P-uptake, P-utilization efficiency, and grain yield under low-P have been identified in WA sorghum and pearl millet, and marker-assisted selection could be possible once these genomic regions are validated. Developing P-efficient genotypes may not, however, be a sustainable solution in itself in the long-term without replenishing the P removed from the system in harvested produce. We therefore propose the use of integrated soil fertility management and systems-oriented management such as enhanced crop-tree-livestock integration in combination with P-use-efficiency-improved varieties. Recycling P from animal bones, human excreta and urine are also possible approaches toward a partially closed and efficient P cycle in WA.

## Introduction

Much interest in food security has focused on depletion of non-renewable energy and land resources. Recently, the depletion of phosphorus (P) is receiving increased interest (Van Vuuren et al., 2010; Cordell and White, 2015) as a major limiting factor. Low soil P is considered to be one of the major constraints for food production in the whole of sub-Saharan Africa (SSA; Verde and Matusso, 2014) and is the main limiting macronutrient for the staple cereal crops of sorghum (*Sorghum bicolor*) and pearl millet (*Pennisetum glaucum*) in the Sahelian and Sudanian regions of West Africa (WA; Bationo and Mokwunye, 1991; Buerkert et al., 2001).

Phosphorus is essential for plant nutrition, serving as a component of DNA and cellular energy transport (Cooper et al., 2011; Obersteiner et al., 2013). The application of P-containing fertilizers is therefore important for food production in the context of P-deficiency. The P delivered by inorganic fertilizers is derived from rock phosphate, which is a non-renewable resource (Cordell et al., 2009; Cooper et al., 2011; Veneklaas et al., 2012). The high transportation and processing costs of inorganic P fertilizer make it generally too expensive and hard to access for many farmers in low-income and food-insecure countries (Obersteiner et al., 2013). A more intensive use of inorganic P fertilizers to increase the largely subsistence-oriented food production in WA is therefore not an option in the foreseeable future (Cordell et al., 2009; Obersteiner et al., 2013). Non-acidulated rock phosphate could be used as a substitute source of P (Bationo and Mokwunye, 1991) but farmers have not widely adopted this technology because it often does not produce visible results in the first year and most rock phosphates are not suitable for direct application. Given these conditions, breeding for crops which may produce higher yields under P-limited conditions appears to make an important contribution to an environmental friendly and economically feasible strategy in order to improve pearl millet and sorghum yields in WA under subsistence farmers’ conditions.

The overall goal of the present article is to explore the prospects of using sorghum and pearl millet genetic diversity to contribute toward solving the P-deficiency issue in WA following a multidisciplinary approach. Specific objectives are to summarize:

(1) The global problem of P scarcity and how it will affect WA small-scale farmers;
(2) Soil P dynamics in WA soils;
(3) Plant responses to P deficiency;
(4) Opportunities to breed for improved crop adaptation to P-limited conditions, with the aspects of existing genetic diversity, the question of direct versus indirect selection, availability of genomic tools to enhance phosphorus use efficiency in crops;
(5) Challenges for improving sorghum and pearl millet adaptation to low-P conditions in WA, including lack of reliable screening procedures for accurate phenotyping and trade-offs between traits of interest; and
(6) Systems approaches to address P deficiency in WA.

## Soil Phosphorus Dynamics

The overall P dynamics in the soil-plant system is a function of the integrating effects of P transformation, availability, and utilization driven by soil, rhizosphere and plant processes (Shen et al., 2011). From a pedo-genetical perspective, soil P originates from primary minerals such as apatite, strengite, variscite, and vivianite (Shen et al., 2011). In magmatic parent rocks the phosphate concentration is generally low (100–300 ppm). Mainly sedimentary processes can lead to accumulation of phosphates that can then be used as mineable resources. However, these processes are rare and most sediments too, are characterized by low P concentrations. These low concentrations are a consequence of the parent material and its weathering (**Table 1**).

**Table 1:** Descriptive statistics of total and plant available phosphorus in topsoil samples of South-West Niger based on Hammer (1994).

	Total P	Plant available P (Bray 1)
		
	mg kg^-1^	mg kg^-1^
Mean	272	7.6
Standard deviation	288	6.6
Min	90	0.5
Max	2110	36.6
*n*	65	53


Through chemical weathering, mainly protolysis, the primary minerals are transformed to soluble orthophosphates. These have different fates: (i) Depending on the ions present in the soil solution (pH-depending), they can react with calcium, iron (Fe), or aluminum (Al) to form secondary minerals. In addition, they can be absorbed to Fe and Al oxyhydrate surfaces and later on be occluded upon further growth of these minerals. Compared to the primary minerals, the secondary minerals have a very low solubility (Blume et al., 2010). (ii) The phosphate anion can be taken up by plants or soil-microorganisms from the soil solution and some of it is then converted to organic P forms, while other parts might be kept as phosphate, stored in the vacuole or active in the cytoplasm (Hinsinger, 2001; Shen et al., 2011; Verde and Matusso, 2014). (iii) Humic (organic matter) substances can also indirectly form complexes with the orthophosphates where the orthophosphates are bound by Al and Fe complexed by the humic substances to form humic-metal-P complexes (HMEP) and this form may account to between 50–80% of P in the soil solution (Gerke, 2010). Organic P mainly exists in form of inositol phosphates, phosphonates, active forms as orthophosphate diesters, labile orthophosphate monoesters, and organic polyphosphates; as a consequence, P occurs in the soil either in inorganic or organic form (Verde and Matusso, 2014). The ratio of both fractions mainly depends on the concentration of organic matter in the soil, and the proportion of inorganic P increases with soil depth.

The inorganic primary and secondary P minerals and the organic P forms (once released from the organism) can become plant available upon chemical reaction (Turner et al., 2002; Condron et al., 2005; Shen et al., 2011). However, this plant available fraction usually represents only several percent of the total P stock. Soils in South-West Niger have on average have plant available P concentrations that are close to the absolute deficiency level (4–5 mg kg^-1^, **Table 1**).

The mobility of inorganic P in most soils is still poorly understood and hardly predictable because of the lack of appropriate methods for studying its speciation and biogeochemical behavior (Hinsinger, 2001). However, P availability in the soil depends on the types and amounts of clay and metal oxides, soil solution pH, ionic strength, concentrations of P and metals (Fe, Al and Ca) and the presence of competing anions, including organic acids (Hinsinger, 2001) as well as symbiotic interaction of the crop plants with micro-organisms (e.g., symbiosis of sorghum/pearl millet with mycorrhiza) that facilitate P uptake.

Phosphorus is taken up by plants either as dihydrogen phosphate ions (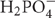) at low soil pH, or as hydrogen phosphate ions (HPO_4_^-2^) at high soil pH, and these occur in soil solutions only at very low concentrations (Raghothama, 1999; Hinsinger, 2001; Hammond et al., 2009). This is the reason why phosphate is leached from soils in only very low amounts under quasi-natural conditions.

Phosphorus acquisition happens by diffusion along the depletion gradient established by the plant and as such is a very slow process. It is therefore important to increase the amount of available P as close to the plant roots as possible in order to increase plant productivity (Hash et al., 2002). A series of responses are triggered in plants under P deficiency which either increase the ability of a plant to acquire P from the soil or the ability of the plant to use the P taken up more efficiently (Vance et al., 2003; Hammond et al., 2004; Jain et al., 2007; Hammond and White, 2008).

### Phosphorus Deficiency in West Africa

Soils in Sahelian WA are either Entisols which are composed of quartz sand or Alfisols which have a clay accumulation horizon and are highly saturated with cations (Kang, 1985), using the soil classification system from United States Department of Agriculture/Natural Resources Conservation Service [USDA/NRCS], 1985). These soils have been shown to have poor structural stability, low water retention, low nutrient holding capacity, low organic matter content, low effective cation exchange and are highly prone to drought (Kang, 1985; Bationo and Mokwunye, 1991). Soils in the sorghum and pearl millet growing areas of WA, the Sudano-Sahelian zone, have been shown to have low total and available P levels with an average total P of 109 mg kg^-1^ and available soil P of sometimes less than 2 mg kg^-1^ (Manu et al., 1991). These soils often have a low capacity to fix P, with sorption data for P in pearl millet growing areas ranging from 27 mg kg^-1^ to 252 mg kg^-1^ (Sanchez and Uehara, 1980). The low plant available soil P in WA can be attributed to several factors: (i) the Aeolian parent materials have low mineral reserves therefore lacking primary minerals for nutrient recycling; (ii) a proportion of P is in occluded form and therefore not available; (iii) low organic matter and the removal of organic residues from the fields (Charreau, 1974). Information on the agronomic practices including combining of organic and inorganic fertilizer, crop rotation, intercropping, among others, for sustainable management of sandy Sahelian soils with regard to P deficiency is provided by Bationo et al. (2007).

## Plant Responses to Phosphorus Deficiency

P-uptake or acquisition efficiency (PAE) and P internal utilization efficiency (PUTIL, sometimes also called PUE) are the two strategies of plant adaptation to P-limited conditions. Phosphorus-uptake efficiency can be defined as the total P in the above-ground plant organs at maturity per unit area. Phosphorus-utilization efficiency is the grain yield per unit of P taken up (Moll et al., 1982; Wang et al., 2010; Manschadi et al., 2014). Both strategies together result in the plants’ P-use efficiency (grain yield per soil available P). Plant adaptations that may contribute toward P-uptake efficiency involve altered root morphology and architecture (Lynch and Brown, 2001; Lynch, 2007), symbioses with vascular arbuscular mycorrhiza (VAM; Smith and Read, 2008) to effectively explore a larger volume of soil, exudation of carboxylates (Lambers et al., 2006, 2011) and secretion of phosphatases (Vance et al., 2003; Richardson et al., 2009) to mobilize organic P forms. Release of citrate and other organic anions into the rhizosphere also helps prevent root damage by chelating aluminum ions (Al^3+^) and lead to more plant available P by mobilizing previously bound P mainly by ligand exchange, dissolution and occupation of P sorption sites (Neumann and Römheld, 1999; Ma et al., 2001). Aluminum-stimulated malic acid secretion encoded by the *Alt1* locus originally identified in wheat by Delhaize et al. (1993) is another mechanism toward Al tolerance with the malic acid mainly secreted from root apices being able to protect seedlings from toxic Al levels. Phosphorus-uptake (acquisition) efficiency resulted in higher yield increase under P-deficient conditions in rice (Vandamme et al., 2016), WA sorghum (Leiser et al., 2014b) and pearl millet (Gemenet et al., 2015b) as compared to internal P-utilization efficiency. However, the higher P-uptake efficiency is expected to result in greater P removal from the cropping system with for example about 1–2 kg P ha^-1^ removed by higher P-uptake rice varieties (Vandamme et al., 2016). These two strategies (P-uptake efficiency and P-utilization efficiency) may be potentially independent and may offer additive benefits if they co-exist in the same genotype (Richardson et al., 2011) and if P-utilization efficiency can be properly separated from the confounding effects of P-uptake efficiency by proper phenotyping approaches (Vandamme et al., 2016; Rose et al., 2016). In WA where soils are characterized by low total P, with low P-fertilizer use, and where residues are removed from the farms (Bationo et al., 2007), combining both P-uptake and P-utilization efficiency would be of particular importance in order to reduce further mining of P from the soils and to enhance adaptation of WA crops to P-limited conditions.

## Opportunities to Breed For Improved Crop Adaptation to Phosphorus Limited Conditions

### Availability of Genetic Variation

The success of a breeding program depends on accessing useful levels of genetic variability for the target trait and an efficient selection method for increasing the frequency of desirable genes or gene combinations. WA sorghums were found to have large genetic variation for P-uptake and utilization efficiencies under P-limited conditions (Leiser et al., 2014b). Differences were observed, however, between specific sorghum germplasm pools. Guinea-race landrace and photoperiod sensitive sorghum varieties, for example showed higher P-uptake efficiency, whereas varieties bred from Caudatum-race introgressed materials, showed higher mean P-utilization efficiency corrected for harvest index (Leiser et al., 2014b). The potential benefits of combining these two pools is suggested by a genotype derived from an inter-pool population that combined superior levels of both P-uptake and utilization efficiency. It was also noted that no single P parameter (P-utilization or P-uptake) trait was appropriate as single selection criterion for enhancing yield under low-P conditions (Leiser et al., 2015).

Pearl millet was also found to possess large genetic variation for P-uptake efficiency, P-utilization efficiency and grain yield performance under P-limited conditions across large-scale regional evaluations in WA under P-deficiency (Gemenet et al., 2014, 2015b). The presence of important genetic variation for these traits in both pearl millet and sorghum indicates that classical breeding can be used to enhance performance under P-deficient conditions in WA.

Symbioses with VAM to enhance access to inorganic forms of P has also been examined in WA pearl millet and sorghum. Beggi (2014) found genetic variation for early VAM colonization and positive correlation of total root length infected with VAM and P-uptake efficiency among open-pollinated pearl millet varieties under low-P conditions. Beneficial effects of VAM infestation in pearl millet were also reported by Bielders et al. (2010) suggesting that this mechanism offers potential for improving P uptake efficiency in WA germplasm. Sorghum, however, although exhibiting significant variation for VAM colonization did not show any useful association between VAM and P-uptake or grain yield under P-limited conditions (Leiser et al., 2016). Breeding for enhanced mycorrhiza colonization in sorghum was therefore concluded to be an ineffective way of enhancing sorghum adaptation to low-P conditions (Leiser et al., 2016).

### Direct versus Indirect Selection for Genotypic Performance under Phosphorus-limited Conditions

A fundamental question for breeders targeting crop improvement for low-P environments is under what conditions selection should be conducted. Indirect selection under high-P conditions may provide higher heritability estimates but may also risk losing genotypes that are best under low-P conditions. Direct selection under low-P conditions on the other hand, may retain these genotypes, only if heritability estimates are not so low as to hinder effective differentiation (Atlin et al., 2001; Bänzinger and Cooper, 2001). Direct selection is expected to be more effective where the genetic correlation between the two contrasting fertility regimes is weak, with significant genotype × P-interactions of cross-over type, and when the broad-sense heritability estimates are similar under both low-P and high-P conditions (Falconer, 1952; Atlin and Frey, 1989). Direct selection under low-P conditions was found to be the strategy of choice for both sorghum (Leiser et al., 2012b) and pearl millet (Gemenet et al., 2014). However, higher error levels did occur under low-P as compared to high-P conditions indicating the importance of using effective experimental designs and statistical analysis methods to minimize error levels. Leiser et al. (2012a) showed that spatial adjustment approaches could reduce residual error and increase heritability under low-P trial conditions and thereby increase efficiency of direct selection in P-limited environments in WA.

### Genomic Tools for Enhancing Adaptation to Low Soil Phosphorus in Crops

Numerous genomic tools offer promising options to applied breeding programs targeting low-P adaptation. The first validated quantitative trait locus (QTL) for P uptake efficiency named *PUP-1* was mapped by Wissuwa et al. (1998, 2002) in rice. Numerous QTLs for low-P adaptation traits such as P-uptake and utilization efficiencies, grain yield under low-P, among others, were subsequently reported in many crops (for a review, please see van de Wiel et al., 2016 and Wissuwa et al., 2016) including sorghum (Leiser et al., 2014a) and pearl millet (Gemenet et al., 2015c). Functional genomics approaches such as transcriptomics and metabolomics have also helped in the discovery of genes involved in adaption to low-P conditions. *PSTOL1* (*phosphorous-starvation tolerance 1*), the underlying gene of PUP-1 (Gamuyao et al., 2012), for example, was discovered through transcriptomics. This gene encodes a protein kinase which enhances early root development in rice thus improving P-uptake efficiency. Homologs of this gene were also identified in sorghum and maize through DNA analysis (Hufnagel et al., 2014; Azevedo et al., 2015). In maize, several genes responsible for root development and morphology have been discovered and a relationship between some of these genes and adaptation to low-P conditions established. The *rootless concerning crown and seminal roots* (RTC) genes encoding a *lateral organs boundaries* (LOB) domain which regulates embryonic seminal and post-embryonic shoot-borne root initiation (Taramino et al., 2007) was for example shown to be overexpressed in a P-efficient line as compared to a P-inefficient line in maize (de Sousa et al., 2012).

Advances in next generation genome sequencing techniques have greatly enhanced the power of gene/QTL discovery and selection through increased molecular marker density and improved resolution (Varshney et al., 2014). These genomic tools offer both increased genetic gain as well as the possibility to expedite the breeding process and reduce its costs. However, the large discrepancy between published results on genomic tools and their actual application in breeding programs has remained a major challenge because most published results are normally not validated for stability across environments and/or genetic backgrounds (Xu and Crouch, 2008). The link between allelic variation of the identified genes and field performance is thus not established in most cases (Wissuwa et al., 2016). With advances in both phenotyping and genotyping technics, however, application of these genomic tools are now becoming possible. The ability to sequence a large number of rice genotypes, for example, together with improved phenotyping for root traits under low-P have allowed precision in estimating trait-allele associations that can be reproduced under field conditions (Wissuwa et al., 2016). There is therefore need to validate the genomic regions associated with performance under P-limited conditions for both sorghum and pearl millet in order to use marker-assisted selection. Proper definition of breeding populations is also needed using the high density marker data from next generation sequencing techniques in order to enhance application of genomics-based selection in sorghum and millet.

## Challenges For Improving Sorghum and Pearl Millet Adaptation to Low Soil Phosphorus Conditions in West Africa

### Lack of Reliable Screening Procedures for Accurate Phenotyping of Specific Adaptation Mechanisms

Most studies into plants’ adaptation to P-limited conditions have mainly dwelled on P-uptake (acquisition) and little has been achieved for P-utilization efficiency. This is mainly because there are no reliable screening methods for P-utilization efficiency because in most cases genotypes used in such studies had different P-uptake capacities which masked the true effects of P-utilization efficiency. Genotypes that were P-uptake inefficient subsequently appeared to be more P-utilization efficient just because they produced relatively more biomass per unit of P taken up as compared to the P-uptake efficient genotypes (Rose et al., 2016). Improving P-utilization efficiency would therefore require evaluation of genotypes with equal P-uptake efficiency. This is still a challenge in a normal breeding program where diverse genotypes are evaluated. P-uptake efficiency is also a function of the root system and its interaction with the rhizosphere. Screening for differences in the root system is a difficult undertaking because roots grow underground and often require destructive sampling. Recovery of the whole root system is therefore difficult and not amenable to evaluation of many genotypes as is normally the case in breeding programs. Controlled conditions using pots, lysimeters, gels and hydroponic systems offer alternative ways of studying root responses to P-deficiency. Screening procedures to estimate the size of plant root systems under controlled conditions (Otani and Ae, 1996; Subbarao et al., 1997a,b; Kaeppler et al., 2000) and field conditions (van Beem et al., 1998; Fenglu and Mugo, 2002) based on non-destructive root capacitance measurements based mainly on models (Dalton, 1995; Dietrich et al., 2012) have been suggested. However, these methods have not been widely applied in practical breeding programs for several reasons. Controlled conditions, though easier to manage, do not wholly represent the conditions under which plants eventually grow under normal field conditions. Recent studies on tolerance to low soil P conditions in WA revealed minimal genetic correlation between controlled pot experiments and field conditions (Gemenet et al., 2015a). Even when carried out in the field, it is difficult to focus solely on root responses to P-deficiency because other stresses occur in combination with low soil P. The effects of soil P-deficiency on pearl millet growth in WA are confounded with drought, the soil physical and chemical environment and possibly unevaluated biological interactions (Gemenet et al., 2015a). Climate variability and change are further challenges to be considered (Haussmann et al., 2012). Differences between genotypic performances under contrasting P-levels were masked in the event of terminal drought stress, which agrees with the findings of Sinclair and Vadez (2002) that P-uptake is reduced to near zero during water stress. Beggi et al. (2015) also found contrasting effects of low soil P on the time to flowering across genotypes, and this would have different effects on yield responses to low soil P in combination with terminal water stress. Al and/or manganese (Mn) toxicities or calcium (Ca), and/or magnesium (Mg) deficiencies may also mask P effects during phenotyping under field conditions. Selecting specifically for low-P adaptation mechanisms alone may therefore prove ineffective for enhancing yield under field conditions. Selection gains for the target population of environments may only be achieved if the selection environments represent the complexity of factors appearing under on-farm field conditions.

### Trade-offs between Adaptation Traits

There are trade-offs between water-uptake (deeper root systems) and P-uptake efficiency-related root traits (shallower foraging root systems; Ho et al., 2005; Lynch, 2011; Manschadi et al., 2014). This is of particular importance for pearl millet in Sahelian regions of WA where drought and low soil P typically overlap. Further research into the genetic variation of crop plants for root systems under field conditions is thus needed to identify genotypes with dimorphic rooting systems capable of both vigorous surface and deep soil horizon root growth (Lynch, 2007; Richardson et al., 2009, 2011).

The carbon costs of more extensive root growth, VAM colonization, or exudation of organic anions for increasing P-uptake, need to be considered (Lynch, 2007; Richardson et al., 2009, 2011; Ryan et al., 2012). The extent of carbon costs may differ by species. Pearl millet for example, showed a positive yield response to early colonization by VAM under low-P conditions (Bielders et al., 2010; Beggi, 2014) whereas sorghum showed a negative response of biomass and no relationship between VAM colonization and final grain yield performance (Leiser et al., 2016).

Increasing P-content of the grain may compromise zinc, iron and calcium bioavailability in human nutrition due to a higher content of phytate, the storage form of phosphorus in the grain, which may also become anti-nutrient (Buerkert et al., 1998; Manschadi et al., 2014). Breeding for lower P concentration in grains has been proposed to increase micronutrient bioavailability and to reduce loss of P from the farming system (Rose et al., 2010, 2016; Leiser et al., 2014b). Pearl millet and sorghum for example were found to partition on average 60 (Gemenet et al., 2015b) and 73% (Leiser et al., 2014b), respectively, of their total P to grain under low-P conditions in WA. Lower P-concentration in rice grain was capable of reducing P removal from the farming system by between 0.5–5 kg P ha^-1^ in the medium to high yield production systems (Vandamme et al., 2016). Reduced P content in grains could be particularly important in WA where soil P deficiency and human micro-nutrient deficiency (“hidden hunger”) are extensive. However, reduction of seed P content can negatively impact germination, seedling establishment and final grain yield under low-P conditions (Raboy, 2009; Robinson et al., 2012; Rose et al., 2012; Vandamme et al., 2016). Lower P concentration in rice grains was, however, not found to affect seedling vigor regardless of soil P status (Pariasca-Tanaka et al., 2015). Also, a selection index that combined low grain-P content and high grain yield under low-P conditions was predicted to be among the most successful for WA sorghum (Leiser et al., 2014b). Targeted selection of reduced phytate P while maintaining the other P forms in the seed would be another option for maintaining Fe and Zn bioavailability. This option was found to reduce germination by 30% but not affect development of the germinated plants (Pilu et al., 2003). Such an approach could be studied further in sorghum and pearl millet. In addition, the effect of reduced P in grains on human nutrition needs to be studied further since P is also the most abundant element in the human body and a substantial amount of it is consumed through cereals (Welch et al., 2009; Rose et al., 2013). In this regard therefore, there seems to be no “one option fits it all” solution and options need to be optimized according to the needs and objectives of a given program.

## Systems Approaches to Addressing the Phosphorus Deficiency Issue in West Africa

The use of P-efficient genotypes provides an opportunity to initially increase crop productivity which may subsequently enable farmers to have surplus income to purchase fertilizer (Lynch, 2007). It will also help address the P-use inefficiencies that commonly occur where P fertilizers are used in large quantities by encouraging reduction in fertilizer use with no reduction in yield (Simpson et al., 2011; Weaver and Wong, 2011). However, genetic advancement of adaptation to low-P soils is not in itself a long-term solution to P deficiency problems in WA and will not offset the need for P input from fertilizer, application of locally available phosphate rock, P placement to seeds at sowing (Buerkert and Hiernaux, 1998) organic matter, manures, or other sources of P to replace P exports (Hash et al., 2002; Sánchez, 2010; Leiser, 2014; Gemenet, 2015). These approaches form part of the integrated soil fertility management (ISFM) practices that have been proposed for SSA and adopted by the Alliance for a Green Revolution in Africa (AGRA; AGRA, 2013). The ISFM approach is defined by Sanginga and Woomer (2009) as the application of soil fertility management practices, together with the knowledge to adapt these to local conditions, in order to maximize fertilizer and organic resource-use efficiency and crop productivity. The ISFM approach links back to farming-systems-oriented research which is an interdisciplinary, integrative, problem-oriented and farmer-centered approach (Darnhofer et al., 2012). Improved crop-livestock integration has been proposed as a strategy toward addressing the challenge of increasing productivity and making African smallholder farming systems more sustainable (Powell and Williams, 1995). In this case, animals produce manure for use in crop production, and crop residues are used as feed and fodder for livestock. Another widely used systems approach involves crop-tree-livestock integration. The extensive ‘agroforestry parklands’ of Sahelian WA involve various tree genera and species grown together with important annual crops in a system shown to provide soil cover that reduces erosion and buffers the impacts of climate change (Bayala et al., 2013). In addition, these trees and shrubs can also provide green fodder that complements crop residues for livestock feeds, firewood, and fruits and leaves for human consumption and for income generation (Zomer et al., 2009; Bayala et al., 2013). Integrated crop-tree-livestock systems adapted to specific local conditions could be maintained as another approach toward a sustainable P management with high internal efficiency. For instance, livestock, especially ruminants, in addition to feeding on crop residues and agro-forestry trees can also harvest P from pasture lands where no crops grow and the resulting manure is then transferred to croplands. Further, P could be recycled from livestock bones e.g., from slaughter houses if proper processing procedures are put in place (Keyzer, 2010). The fact that most of the P is removed from farming systems in the form of grain, coupled with the increasing rural-urban migration prevalent in the whole of SSA (Hove et al., 2013) imply that most P is lost as human waste in cities (Keyzer, 2010). However, it is possible to either directly recycle wastewater in urban agriculture or to extract P from urban wastewaters in the form of the mineral struvite (MgNH_4_PO_4_*6H_2_O) that then can be used as fertilizer in agriculture (Le Corre et al., 2009). It is therefore necessary to put in place policies that will enhance P recovery from human excreta from cities and return it to farming systems as an approach toward closing the human P cycle (Childers et al., 2011). MacDonald et al. (2011) also proposed a global P fertilizer use strategy and reduction of P fertilizer use in areas with intense P surpluses and redistribution of such fertilizer to P-deficit croplands as another approach to reduce global P imbalances.

Farmers, researchers and other development partners operate within certain government legislative frameworks. In order to address the P deficiency issue in WA, governments within the region need to commit to sustainably reduce poverty and improve food security. The governments for example need to increase public investment in research and development activities; encourage private sector investments in agriculture through enabling policies; facilitate the generation and sharing of relevant new scientific knowledge (AGRA, 2013); facilitate breeding and improve seed systems of low-P adapted varieties (AGRA, 2014), among other commitments for example as made by SSA governments in the Maputo declaration of 2003.

Rock phosphate is a finite resource over which geopolitical tension may arise (Cordell et al., 2009), especially with the increasing global human population and food insecurity in WA (FAO et al., 2013). Tackling the issue of low-P in WA sorghum and pearl millet production systems will therefore contribute to prosperity and peace, but will require action by all players, from smallholder farmers to researchers, non-governmental organizations, international development partners and policy makers.

## Author Contributions

All authors are part of an interdisciplinary research team that jointly implemented the reported research and developed the manuscript. DG, WL, FB, VV, CH, HR, and EW conducted the research, BH, LH, AB, HR, EW, CH, and VV designed and advised on the research, data collection, analysis and interpretation of results, DG wrote first draft of manuscript; all others reviewed and contributed to manuscript finalization.

## Conflict of Interest Statement

The authors declare that the research was conducted in the absence of any commercial or financial relationships that could be construed as a potential conflict of interest.
